# Exosomal MicroRNA-181a Derived From Mesenchymal Stem Cells Improves Gut Microbiota Composition, Barrier Function, and Inflammatory Status in an Experimental Colitis Model

**DOI:** 10.3389/fmed.2021.660614

**Published:** 2021-06-24

**Authors:** Li Gu, Feng Ren, Xianrui Fang, Lianwen Yuan, Ganglei Liu, Shalong Wang

**Affiliations:** ^1^Department of Gastroenterology, The Second Xiangya Hospital of Central South University, Changsha, China; ^2^Department of General Surgery, The Second Xiangya Hospital of Central South University, Changsha, China; ^3^Department of Surgery, Shandong Laiyang Health School, Laiyang, China

**Keywords:** ulcerative colitis, exosomal microRNA-181a, gut microbiota, intestinal barrier function, mesenchymal stem cells

## Abstract

**Background:** Mesenchymal stem cell (MSC)-derived exosomes (Exos) are recently proved to be a promising candidate for ulcerative colitis (UC), but the mechanism remains unclear. We investigated the effects of MSC-derived exosomal microRNA-181a (miR-181a) on gut microbiota, immune responses, and intestinal barrier function in UC.

**Methods:** Human bone marrow MSC-derived Exos were extracted and identified *via* transmission electron microscopy (TEM), Nanoparticle Tracking Analysis (NTA), and Western blotting. Dextran sodium sulfate (DSS)-induced colitis model and lipopolysaccharide (LPS)-induced human colonic epithelial cell (HCOEPIC) model were established to determine the effect of MSC-Exos on gut microbiota, immune responses, and intestinal barrier function *in vivo* and *in vitro*. The relationship between miR-181a and UC was analyzed using the Gene Expression Omnibus (GEO) database. MSC-miR-181-inhibitor was used to reveal the role of exosomal miR-181a in DSS-induced colitis.

**Results:** TEM and NTA results showed that Exos of a diameter of about 100 nm with the round and oval vesicle-like structure were successfully extracted. The expressions of the CD63, CD81, and TSG101 proteins were positive in these Exos. After MSC-Exo treatment, the colon length in colitis mice increased; colon inflammatory injury decreased; TNF-α, IL-6, IL-1β, IL-17, and IL-18 levels decreased; and Claudin-1, ZO-1, and IκB levels increased. In addition, the structure of the gut microbiota in DSS-induced colitis mice was changed by MSC-Exos. MSC-Exos showed antiapoptotic effects on LPS-induced HCOEPIC. The protective effects decreased significantly by treatment with MSC-Exos interfered with miR-181a inhibitor *in vivo* and *in vitro*.

**Conclusion:** MSC-derived exosomal miR-181a could alleviate experimental colitis by promoting intestinal barrier function. It exerted anti-inflammatory function and affected the gut microbiota. This indicated that MSC exosomal miR-181a may exhibit potential as a disease-modifying drug for UC.

## Introduction

Ulcerative colitis (UC) is a chronic, idiopathic inflammatory bowel disorder of the colon. It is characterized by relapsing and remitting continuous mucosal inflammation, extending from the rectum to the more proximal colon (1). The overall incidence and prevalence of UC have been increasing over time worldwide, resulting in substantial patient burden and costs to healthcare systems (2–4). There is still no medical or surgical cure for UC. Although immunosuppression has been demonstrated to be highly effective in UC, a subset of patients remain refractory to conventional therapies regarding symptom management and mucosal healing (5). Moreover, immunosuppression is often associated with side effects including increased risk of developing opportunistic infections, skin cancer, lymphoma, and neurological (6, 7). Thus, a clear unmet need persists for novel and well-tolerated therapeutic approaches for UC.

Mesenchymal stem cells (MSCs) are recently proved to be a promising candidate for the treatment of immune and inflammatory disorders, due to their potent immunomodulatory properties and tissue repair capacity (8). Several experimental and clinical studies have demonstrated that MSCs can ameliorate intestinal inflammation in UC (9, 10). However, the clinical application of MSCs transplantation still faces safety concerns, with potential unwanted effects of MSC-based cell therapy (11).

Exosomes (Exos) are extracellular vesicles (EVs) with an average diameter of about 100 nm, containing complex RNAs and proteins. The exchange of active ingredients between cells can be achieved through Exos (12). MSCs are considered to exert their beneficial effects primarily through their paracrine factors of the EVs including Exos (13). In addition, compared with their parent cells, MSC-derived Exos (MSC-Exos) confer several advantages such as lower immunogenicity, capacity to cross biological barriers, and fewer safety concerns (11). Thus, MSC-Exos may be potentially used to develop a cell-free Exo-based therapy as an alternative to MSC-based therapy.

Recently, MSC-Exos have demonstrated efficacy for UC in animal studies (14, 15). However, the underlying mechanisms remain elusive. Further experimental studies need to be done to unveil the influence of MSC-Exos on major related pathways in UC, such as immune responses, intestinal barrier functions, and gut microbiota. Moreover, Exos contain various biological components such as nucleic acids, proteins, and other components like lipids (16). It is significant to identify the exact specific MSC-Exo-contained molecules responsible for therapeutic effects for UC.

MicroRNA-181a (miR-181a) is a member of the miR-181 family. Several reports have shown that miR-181a can regulate both innate and adaptive immune responses (17, 18). Meanwhile, exosomal miR-181a plays an important role in regulating endoplasmic reticulum stress (ERS), muscle atrophy, and apoptosis (19). In light of the previous findings, we conducted this study to further verify the effects of MSC-derived exosomal miR-181a in UC and to explore the mechanisms of miR-181a in UC from the aspect of gut microbiota, immune responses, and intestinal barrier function.

## Materials and Methods

### Cell Culture

Human bone marrow MSCs (ZQ0266, Shanghai Zhongqiaoxinzhou Biotech, Shanghai, China) were cultured in Dulbecco's modified Eagle's medium (DMEM)/F-12 medium (R8758, Sigma, St. Louis, MO, USA) containing 10% fetal bovine serum (FBS) and 1% penicillin-streptomycin (SV30010, Beyotime Biotechnology, Shanghai, China). Human colonic epithelial cells (HCOEPICs; 2950, Shanghai Zhongqiaoxinzhou Biotech) were cultured in HCOEPIC Medium (2951, Shanghai Zhongqiaoxinzhou Biotech). Lipopolysaccharide (LPS; 10 μg/ml, 24 h) was used to stimulate the MSCs and HCOEPICs.

### Extraction and Uptake of Mesenchymal Stem Cell-Derived Exosomes

Exos were extracted from the MSC culture supernatant. Firstly, MSCs were divided into three groups, including a phosphate-buffered saline (PBS) group, an Exo group, and a LPS-Exos. The PBS group was a negative control without Exos. The Exo group and LPS-Exos were Exo extracted from MSCs that are not stimulated by LPS and stimulated *via* LPS (10 μg/ml, 24 h), respectively. To verify transfection efficiency, MSCs were divided into another three groups, containing the Exo group, an inhibitor-negative control (inhi-NC), and an inhibitor group. MSCs in the Exo group were treated the same way as before. MSCs in the inhi-NC and inhibitor groups were transfected with 100 nM scrambled sequences and inhibitor using Lipofectamine 2000 (Thermo Fisher Scientific, Waltham, MA, USA) complexes, respectively. After a predetermined time, the Exos were isolated from the aforementioned MSCs. An Exo extraction kit (EXOQ5A-1, SBI, Palo Alto, CA, USA) was used according to the manufacturer's instructions. MSCs were cultured in serum-free DMEM. The supernatant was collected for 48–72 h, and Exos were extracted for subsequent detection. The extracted Exos were resuspended in PBS in an appropriate volume. The morphology and size of Exos were analyzed *via* transmission electron microscopy (TEM). The diameter of exosomal particles was further detected *via* Nanoparticle Tracking Analysis (NTA). Exos were labeled with live-cell fuel PKH26 (PKH26PCL, Sigma) and then cocultured with HCOEPICs for 12 h. The fluorescence of each group was observed by laser scanning confocal microscopy (LSCM). In the control group, the HCOEPICs were treated with the same volume of PBS as the Exo group for 12 h.

### Mesenchymal Stem Cell-Derived Exosome Treatment in the Dextran Sodium Sulfate Model

Forty healthy C57BL/6 mice (6–8 weeks old) were purchased from Hunan SJA Laboratory Animal Co., Ltd. (Hunan, China) and randomly divided into four groups (*n* = 10): (i) a control group (Control), (ii) a dextran sodium sulfate DSS group, (iii) an MSC-Exo group, and (iv) an LPS-MSC-Exo group. Mice with DSS treatment received 4% DSS in drinking water on days 3–9 to establish the DSS model. The MSC-Exos and LPS-MSC-Exo groups were treated with DSS in water and Exos (32 mg/kg) *via* intravenous injection on days 1, 3, 5, and 7, which were extracted from MSCs without or with LPS stimulation, respectively (20). On the last day of administration, the mice were anesthetized with 2% pentobarbital sodium (30 mg/kg). All the mice were given free access to water and food and were kept in an environment with a constant room temperature (25 ± 2°C), 55% humidity, and a 12/12-h light–dark cycle.

### Mesenchymal Stem Cell-Derived Exosome Treatment of Human Colonic Epithelial Cells

Next, the HCOEPICs were divided into four groups according to different processing methods: (i) a control group, (ii) an LPS group, (iii) an MSC-Exo group, and (iv) an LPS-MSC-Exo group *in vitro*. The cells in the control group were cultured normally. The cells in the LPS group were treated with LPS (10 μg/ml, 24 h) (21, 22). Furthermore, under LPS stimulation, the cells in the MSC-Exos and LPS-MSC-Exo groups were treated with Exos, which were extracted from MSCs without or with LPS stimulation for 24 h, respectively.

### Mesenchymal Stem Cell–microRNA-181-Inhibitor Treatment *in vivo* and *in vitro*

To further determine the effect of miR-181a in MSC-Exos on experimental colitis, 40 mice were randomly divided into four groups: (i) a control group, (ii) a DSS group, (iii) an MSC-con group, and (iv) an MSC-miR-181-inhibitor group (MSC-miR-181-inhi). Mice in the control and DSS groups were treated as above, respectively. The MSC-miR-181-inhibitor group was injected with Exos (32 mg/kg) extracted from MSCs that were transfected with miR-181-inhibitor. The MSC-con group was treated with Exos (32 mg/kg) extracted from MSCs that were transfected with negative control.

Then, *in vitro* assays were performed. The HCOEPICs were divided into another four groups according to different processing methods: (i) a control group, (ii) an LPS group, (iii) an MSC-con group, and (iv) an MSC-miR-181a-inhibitor group. The control and DSS groups were treated as described earlier. The cells in the MSC-con and MSC-miR-181a-inhibitor groups were treated with Exos derived from transfected cells under LPS stimulation. Next, the cells were collected for follow-up experiments.

### H&E Staining

After 4 h of fixation with 4% paraformaldehyde, the colonic tissues of mice were dehydrated, embedded, and sliced. After H&E staining, a microscope (BA210T, Motic, Kowloon, Hong Kong) was used to observe the pathological structure of the colon.

### TUNEL Staining

Colonic tissues were sectioned with paraffin wax, and apoptosis was detected using a TUNEL kit (40306ES50, Yeasen, Shanghai, China) according to the manufacturer's instructions. Tissue apoptosis was observed *via* fluorescence microscopy. Three fields were randomly selected to calculate the number of TUNEL-positive cells.

### ELISA Kits

Serum and colonic TNF-α (CSB-E04741m, CusaBio, Wuhan, China), IL-6 (CSB-E04639m, CusaBio, China), IL-1β (CSB-E08054m, CusaBio, China), IL-17 (CSB-E04608m, CusaBio, China), IL-18 (CSB-E04609m, CusaBio, China), and myeloperoxidase (MPO) (A044-1-1, NanJing JianCheng Bioengineering Institute, Nanjing, China) quantitative ELISA kits were used according to the instructions. All experiments were repeated three times in each group.

### Flow Cytometry Assay

To analyze HCOEPIC apoptosis, an Annexin V-FITC apoptosis detection kit (KGA108, KeyGEN, Nanjing, China) was used. According to the manufacturer's instructions, 500 μl of binding buffer was added to suspend the HCOEPICs, and then 5 μl of Annexin V-FITC was added. After mixing, 5 μl of propidium iodide was added and incubated with the cells at room temperature for 15 min. Cell apoptosis was analyzed *via* flow cytometry (A00-1-1102, Beckman, Brea, California, USA). The cells in the different groups were treated with 10 μM of DCFH-DA (mother liquid concentration: 10 mM) and incubated at 37°C for 20 min. The cells were washed three times with a serum-free medium and digested with trypsin. After cell collection, flow cytometry (A00-1-1102, Beckman) was used to detect reactive oxygen species (ROS) accumulation.

### Cell Counting Kit 8 Assay

HCOEPICs in the logarithmic growth phase were digested and counted. The cells were seeded in 96-well-plates at a density of 1 × 10^4^ cells/well (100 μl/well). A 10% Cell Counting Kit 8 (CCK-8) solution (NU679, DOJINDO, Kumamoto, Japan) was added to each well and incubated with the cells at 37°C for 4 h. The absorbance was measured at 450 nm using a Bio-Tek microplate (MB-530, Heales, Shenzhen, China).

### Western Blotting

Total protein was from the mouse colonic tissue or HCOEPICs. The extracted protein in each group was quantified using a bicinchoninic acid (BCA) protein concentration determination kit (CB86198073, Chemical Book). The expression levels of CD81, CD63, TSG101, Claudin-1, ZO-1, TNF-α, IκB, Caspase-3, Bax, and Bcl-2 were detected by Western blotting. After blocking, the membranes were incubated with antibodies against CD81 (ab109201, 1:4,000, Abcam, Cambridge, UK), CD63 (25682-1-AP, 1:1,000, ProteinTech, Rosemont, IL, USA), TSG101 (14497-1-AP, 1:2,000, ProteinTech), Claudin-1 (1:4,000, 13050-1-AP, ProteinTech), ZO-1 (1:8,000, 21773-1-AP, ProteinTech), TNF-α (1:600, 17590-1-AP, ProteinTech), IκB (1:2,000, 10268-1-AP, ProteinTech), Caspase-3 (1:500, 19677-1-AP, ProteinTech), Bax (1:6,000, 50599-2-Ig, ProteinTech), and Bcl-2 (1:2,000, 12789-1-AP, ProteinTech) overnight at 4°C. The membranes were then rinsed three times with PBS-T and incubated with anti-rabbit IgG (1:6,000, SA00001-2, ProteinTech) and anti-mouse IgG (1:5,000, SA00001-1, ProteinTech) antibodies at room temperature for 1.5 h. After enhanced chemiluminescence (ECL) exposure, an Odyssey infrared imaging system (Li-Cor Biosciences, Lincoln, NE, USA) was used to detect the protein bands. β-Actin (1:5,000, 66009-1-Ig, ProteinTech) was used as the internal reference.

### Quantitative Real-Time PCR

Total RNA was extracted from mouse colonic tissue, HCOEPICs, and Exos after treatment with TRIzol reagent (Thermo, USA). Total mRNA was used as a template to reverse transcribe cDNA. β-Actin was used as an internal reference to analyze the expression of miR-181a and mRNAs. The specific primers used for qRT-PCR were as follows: miR-181a-F, AACATTCAACGCTGTCGGTG, and miR-181a-R, RAGCCATAGGGTACAATCAAC-GGG; TNF-α-F, GAACCCCGAGTGACAAGCCT, and TNF-α-R, TATCTCTCAGCT-CCACGCCAT; IκB-F, CAAATCTTTCAGGGCAGAGTCCGA, and IκB-R, TGCCGAA-AAGAGGACAACCAC; β-actin-F, ACCCTGAAGTACCCCATCGAG, and β-actin-R, AGCACAGCCTGGATAGCAAC. PCR amplification was commonly used. The 2^−Δ*ΔCt*^ method was used to measure the RNA expression levels.

### Bioinformatics Analysis

Data were obtained from the Gene Expression Omnibus (GEO) database research cohort GSE68306. The samples were divided into a UC01 patient group (*n* = 20), UC02 patient group (*n* = 9), and normal control group (*n* = 16). The data type was miRNA chip. Differential miRNA expression was analyzed with the R package limma in the UC01 vs. normal and UC02 vs. normal groups. The screening criteria were |logFC| > 1 and *P* < 0.05. The R package heatmap was used to cluster the expression patterns of differentially expressed miRNAs in the two groups of samples, and heat maps of the two groups were drawn to screen the significantly low-expression miRNAs in the UC samples. Pearson correlation coefficient analysis was performed between the mRNA microarray data of GSE68306 and the selected differentially expressed miRNAs. The screening criterion was |Pearson correlation coefficient| > 0.75. Then, a coexpression network was constructed using Cytoscape software. The R package ClusterProfiler was used to annotate the Gene Ontology (GO) functions of differentially expressed mRNAs in the coexpression network, and the significantly enriched pathways were plotted.

### Immunofluorescence Staining

Paraffin-embedded tissues were divided into four groups. After paraffin sections were dewaxed to water, antigen was heat repaired, endogenous enzymes were inactivated, and the tissues were incubated with primary antibodies against Claudin-1 (1:50, 13050-1-AP, ProteinTech) and ZO-1 (1:50, 21773-1-AP, ProteinTech). After being washed with PBS, the tissues were incubated with secondary antibody for 30 min. Then, DAPI solution was used to stain the nuclei at 37°C. Images were collected and analyzed using fluorescence microscopy and Image-Pro Plus (IPP) software (Media Cybernetics, Inc., Rockville, MD, USA).

### DNA Extraction and High-Throughput Sequencing

According to the manufacturer's instructions, a fecal genomic DNA Kit (#DP328-02, TIANGEN, Beijing, China) was used to extract DNA. A dsDNA HS Assay Kit (12640ES76, Yeasen) was used to detect the concentration of extracted DNA. After amplification and purification, the whole genome of the sample was sequenced on an Illumina NovaSeq platform. After the original data for quality control were obtained, the species composition of the samples was analyzed by comparison with the Silva-132-99 database. QIIME 2 (QIIME 2-2020.2) and R software 4.0.2 were used for sequence data analysis.

### Statistical Analysis

Statistical analysis was performed using GraphPad Prism 8 Software (GraphPad Software, Inc., La Jolla, CA, USA). All data are presented as the mean ± standard error of mean (SEM). Differences between two or more groups were analyzed using unpaired *t*-tests or one-way analysis of variance. *P* < 0.05 indicated a significant difference.

## Results

### Validation of Mesenchymal Stem Cell-Derived Exosomes

To identify MSC-Exos, the round and oval vesicle-like structure of Exos was observed *via* TEM (Supplementary Figure 1A). NTA results showed that the exosomal particle diameter was concentrated at about 100 nm (Supplementary Figure 1B). Western blotting analysis showed that the expression levels of TSG101, CD81, and CD63 in Exos were higher in the LPS-MSC-Exo group than in the MSC-Exo group (Supplementary Figure 1C). To verify the ability of HCOEPICs to absorb Exos, an absorbance assay was performed. The control, in which PBS was added instead of MSC-Exos, did not show red fluorescence under LSCM. When HCOEPICs were cocultured with Exos (the Exo group), a large number of red vesicles gradually infiltrated the cells. Moreover, most of these proteins were concentrated in the cytoplasm (Supplementary Figure 1D). qRT-PCR results indicated that miR-181a was upregulated in Exos with and without LPS stimulation compared with the control group, and miR-181a was more highly expressed in Exos with LPS stimulation (Supplementary Figure 1E). These results indicate that HCOEPIC can effectively absorb Exos. Further, MSCs were transfected with miR-181a inhibitor. In the MSC-Exos miR-181a inhibitor group (inhibitor), the expression level of miR-181a was significantly reduced (Supplementary Figure 1F). These results indicate that we successfully extracted Exos from MSCs and that HCOEPIC effectively absorbed Exos. Additionally, the expression level of miR-181a in the Exos was noteworthy.

### Mesenchymal Stem Cell-Derived Exosome and Lipopolysaccharide–Mesenchymal Stem Cell-Derived Exosomes Alleviated Dextran Sodium Sulfate-Induced Colitis *in vivo*

**Figure 1:**
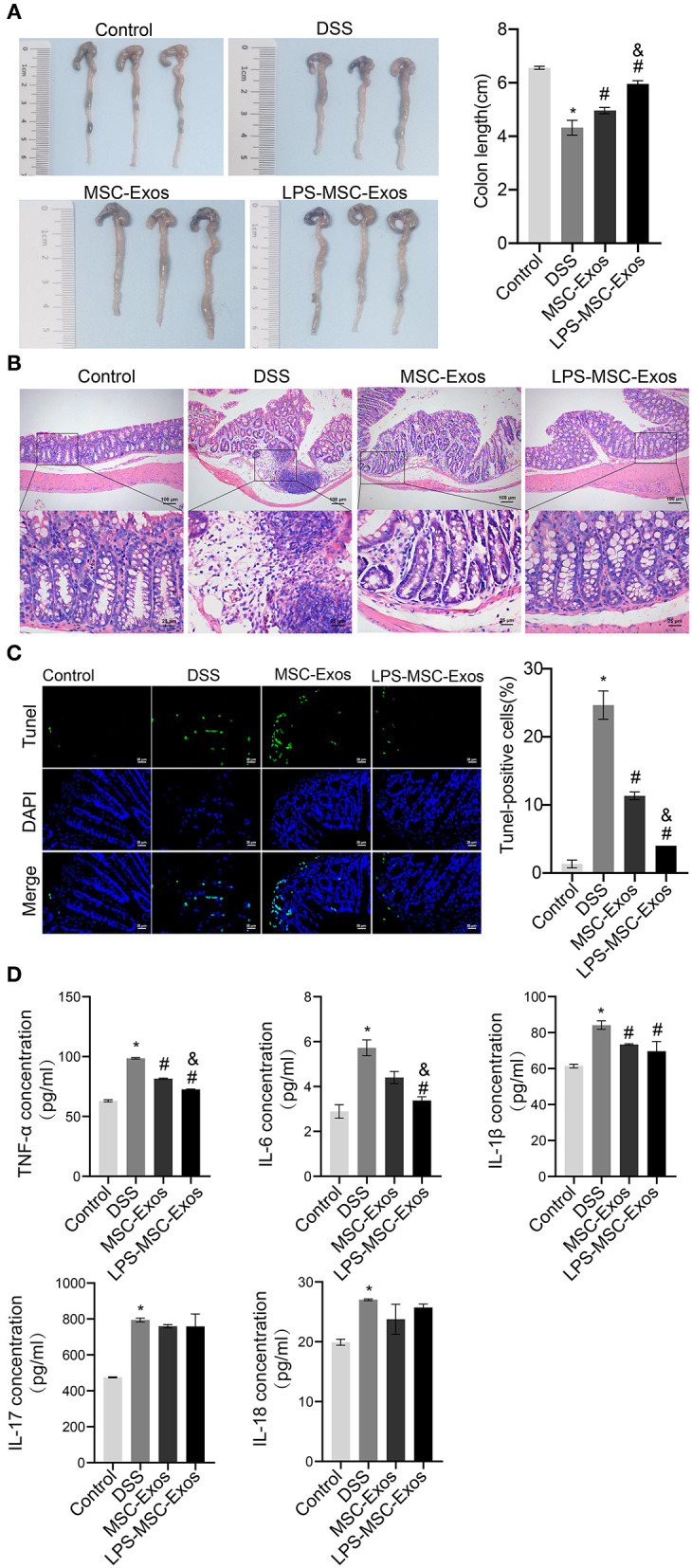
MSC-Exos and LPS-MSC-Exos alleviated DSS-induced colitis *in vivo*. **(A)** The appearance and length of the colon in mice were analyzed. **(B)** H&E staining of colonic pathological features in the mice. Scale bar: 100 or 25 μm. **(C)** A TUNEL assay was used to detect cell apoptosis in colonic tissue from the mice. Scale bar: 25 μm. **(D)** The concentrations of TNF-α, IL-6, IL-1β, IL-17, and IL-18 were measured *via* ELISA. *P < 0.05 vs. the control group. ^#^*P* < 0.05 vs. the DSS group. ^&^*P* < 0.05 vs. the MSC-Exo group. *n* = 10 mice/group. MSC, mesenchymal stem cell; Exos, exosomes; LPS, lipopolysaccharide; DSS, dextran sodium sulfate.

We established a DSS-induced colitis mouse model to investigate the effects of MSC-Exos on experimental colitis. Compared with that in the control group, the colon length in the DSS model group was shorter, which was significantly reversed in the MSC-Exos and LPS-MSC-Exo groups (Figure 1A). The results of H&E and TUNEL staining indicated that DSS treatment induced colonic injury, while the colon from the MSC-Exos and LPS-MSC-Exo groups showed less inflammatory cell infiltration, better structural integrity, and less apoptosis (Figures 1B,C). The expression of inflammatory cytokines in serum, including TNF-α, IL-6, IL-1β, IL-17, and IL-18, in the DSS group, was increased. In contrast, the serum inflammatory cytokines in MSC-exo-treated and LPS-MSC-exo-treated mice were decreased. Specifically, significant changes were observed in the expression of TNF-α, IL-6, and IL-1β. After MSC-Exo treatment, the expression levels of IL-1β, IL-17, and IL-18 had a decreasing trend (Figure 1D). These data suggested that both MSC-Exos and LPS-MSC-Exos played a protective role during colon inflammatory injury in DSS-induced colitis. In addition, LPS-MSC-Exos might be more efficient than MSC-Exos.

### Mesenchymal Stem Cell-Derived Exosomes and Lipopolysaccharide–Mesenchymal Stem Cell-Derived Exosomes Prevented Lipopolysaccharide-Induced Injury *in vitro*

**Figure 2:**
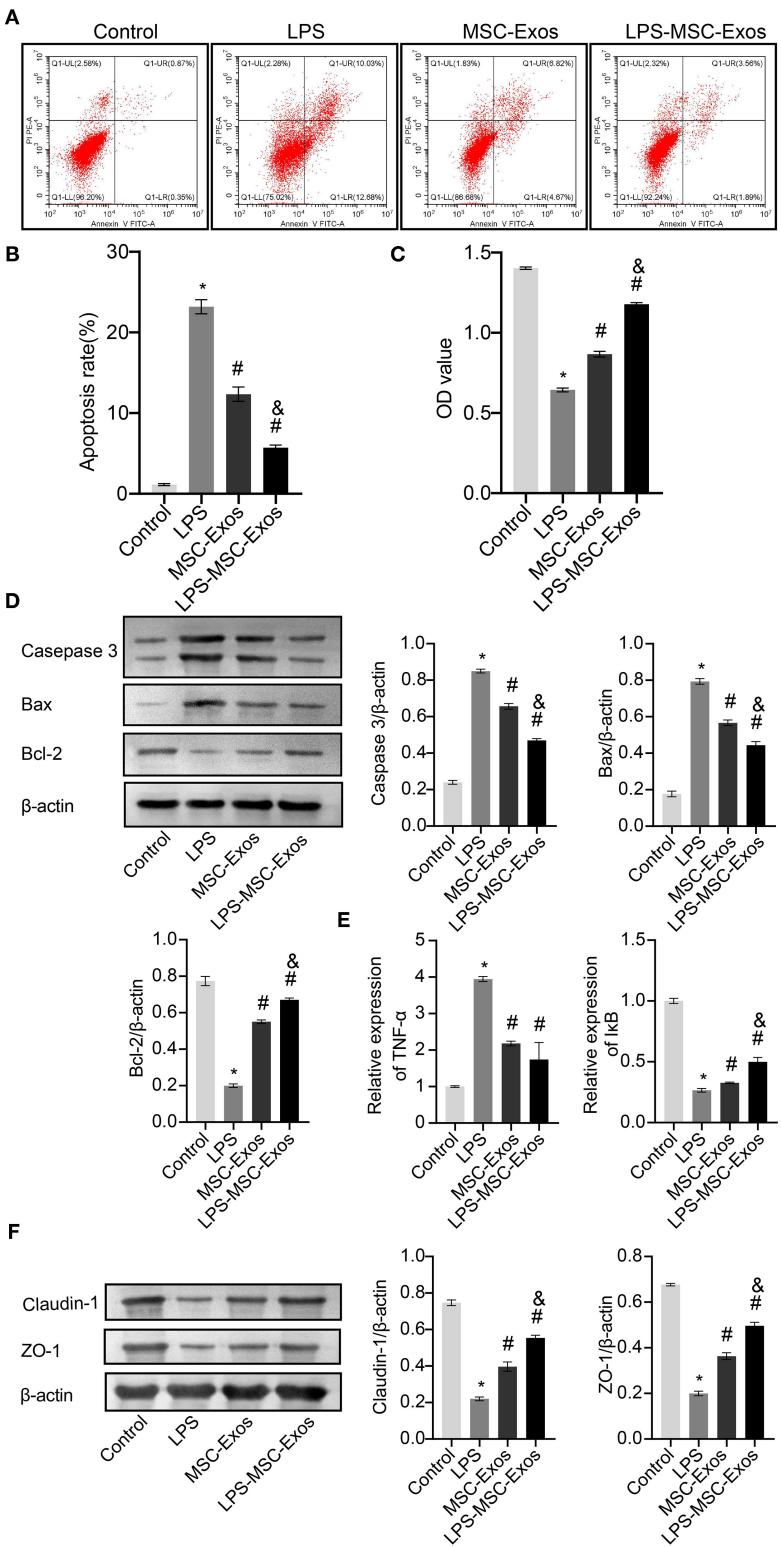
MSC-Exos and LPS-MSC-Exos prevented LPS-induced injury *in vitro*. **(A,B)** Apoptosis was detected by flow cytometry. **(C)** CCK-8 assays were used to detect the viability of HCOEPICs. **(D)** Western blotting was performed to detect the expression of Caspase-3, Bax, and Bcl-2 in HCOEPICs. **(E)** qRT-PCR was used to detect the expression levels of TNF-α and IκB in HCOEPICs. **(F)** The expression levels of Claudin-1 and ZO-1 were detected *via* Western blotting. *P < 0.05 vs. the control. ^#^*P* < 0.05 vs. the LPS group. ^&^*P* < 0.05 vs. the MSC-Exo group. MSC, mesenchymal stem cell; Exos, exosomes; LPS, lipopolysaccharide; DSS, dextran sodium sulfate; CCK-8, Cell Counting Kit 8; HCOEPICs, human colonic epithelial cells.

Next, we investigated whether MSC-Exos have a direct effect on HCOEPICs. Flow cytometry and CCK-8 assay results revealed that the cells in the MSC-Exos and LPS-MSC-Exo groups showed markedly rescued apoptosis and viability after LPS-induced injury (Figures 2A–C). To further determine the antiapoptotic effects of MSC-Exos, the expression of apoptosis-related proteins, including Caspase-3, Bax, and Bcl-2, were detected. The results demonstrated that the expression levels of Caspase-3 and Bax were increased in the LPS group compared with the control group. However, their expression levels were significantly decreased in the MSC-Exos and LPS-MSC-Exo groups compared with the LPS group. Bcl-2 showed the opposite pattern (Figure 2D). Together, the MSC-Exos contribute to protection against LPS-induced injury. More importantly, the Exos derived from LPS-induced MSCs might be more efficient. Furthermore, to determine the effect of TNF-α signaling in MSC-Exos on LPS-induced injury, qRT-PCR was performed. The results showed that the relative TNF-α expression was downregulated in the MSC-Exos and the LPS-MSC-Exo groups, while IκB expression was upregulated compared with that in the LPS group (Figure 2E). Moreover, Western blotting results showed that the expression levels of tight junction proteins, including Claudin-1 and ZO-1, were increased in the MSC-Exos and LPS-MSC-Exo groups compared with the LPS group (Figure 2F). These results indicate that the MSC-Exo group showed a good therapeutic effect in reversing LPS-induced injury *in vitro*. Specifically, the LPS-MSC-Exo group showed a better therapeutic effect. These results were consistent with those of the *in vivo* experiments.

### MicroRNA-181a Expression Levels Were Downregulated in Ulcerative Colitis Patients

**Figure 3:**
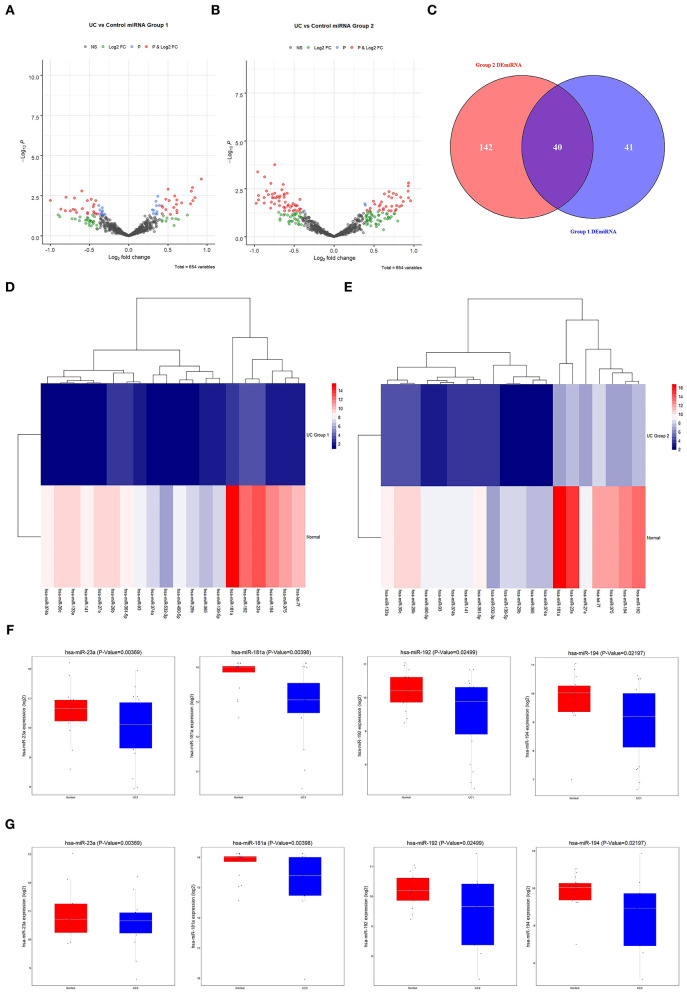
Screening of differentially expressed miRNAs associated with UC. **(A)** The GEO database was used to analyze the expression of differentially expressed miRNAs in Group 1. **(B)** The GEO database was used to analyze the expression of differentially expressed miRNAs in Group 2. **(C)** Venn diagram of 40 candidate miRNAs. **(D,E)** Heat maps of 20 candidate miRNAs. **(F,G)** The expression levels of miR-23a, miR-181a, miR-192, and miR-194 in UC in Group 1 and 2 were analyzed using the GEO database. All were statistically significant. UC, ulcerative colitis; GEO, Gene Expression Omnibus.

Differential expression analysis of miRNAs associated with UC in normal and diseased samples showed differentially expressed miRNAs in Group 1 and 2 (volcano plot in Figures 3A,B). Forty differentially expressed miRNAs were shared by the two groups. Of these, 28 were downregulated and 12 were upregulated (Figure 3C). The clustering heat map showed the top 20 differentially expressed miRNAs that were significantly downregulated in Group 1 and 2 (Figures 3D,E). Box plots indicated the top four differentially expressed miRNAs decreased in both Group 1 and 2 (Figures 3F,G): miR-23a, miR-181a, miR-192, and miR-194. Of these, miR-181a was the most significantly different. Therefore, we focused on the regulatory mechanism of miR-181a in UC.

**Figure 4:**
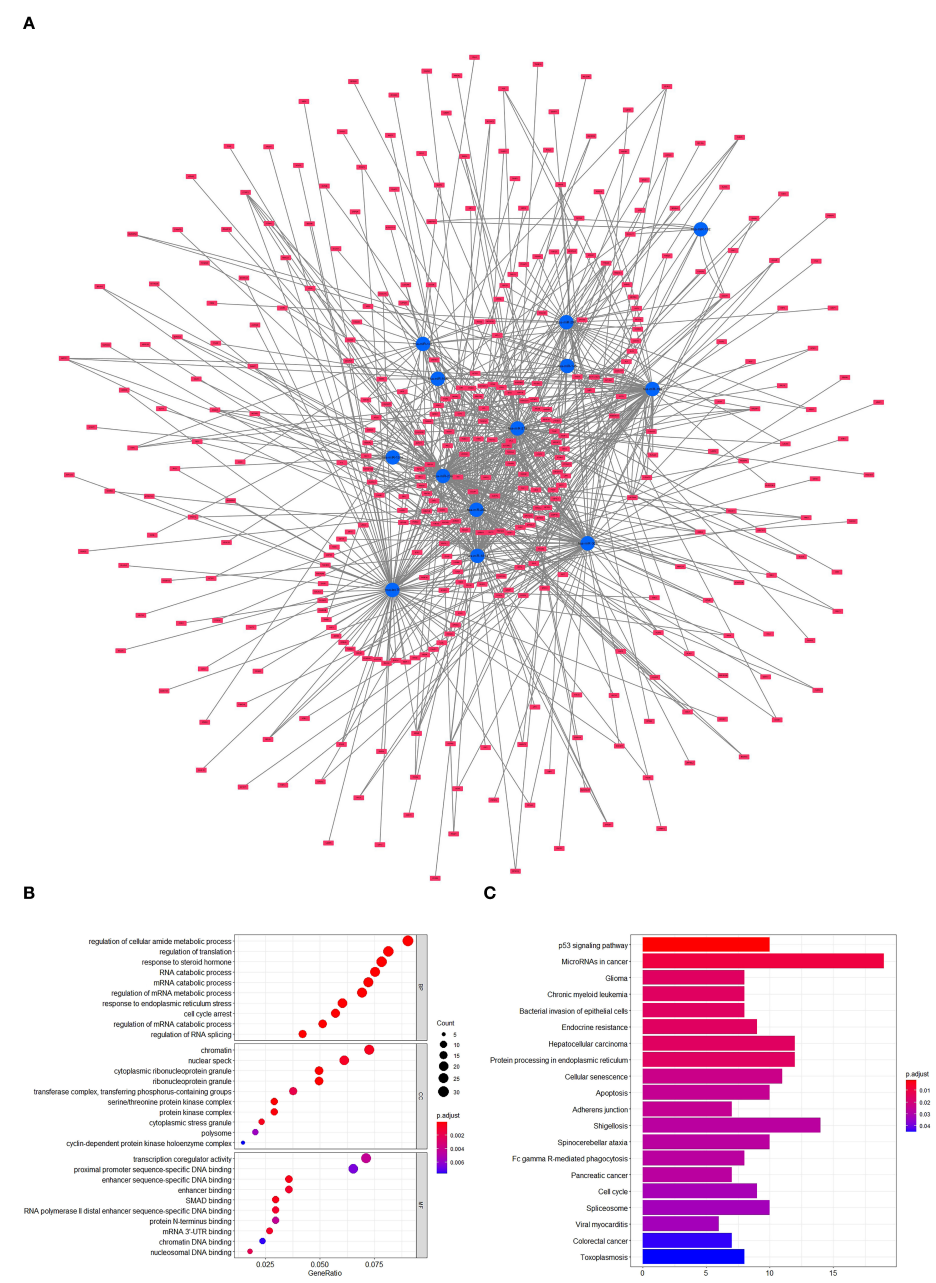
Prediction and analysis of related genes and pathways. **(A)** Coexpression network of differentially expressed miRNAs and mRNAs. **(B)** GO analysis of miR-181a. **(C)** KEGG pathway analysis of miR-181a. GO, Gene Ontology; KEGG, Kyoto Encyclopedia of Genes and Genomes.

To forecast the potential biological function of miR-181a, we analyzed the coexpression network of differentially expressed miRNAs and mRNAs. As shown in Figure 4A, the blue circular nodes represent miRNAs, a total of 13 miRNAs, which were all significantly downregulated in patients with UC. The red rectangular nodes represent genes regulated by differentially expressed miRNAs (Figure 4A). In addition, GO and Kyoto Encyclopedia of Genes and Genomes (KEGG) enrichment analyses of miR-181a were performed. Significant GO annotation included BP (regulation of cellular amide metabolic process, regulation of translation, and response to steroid hormone), CC (chromatin, nuclear speck, and cytoplasmic ribonucleoprotein granule), and MF (transcription coregulator activity, proximal promoter sequence-specific DNA binding, and enhancer sequence-specific DNA binding) (Figure 4B). The main KEGG pathways were involved in miRNAs in cancer, glioma, and the p53 signaling pathway (Figure 4C).

### Exosomal microRNA-181a Attenuated Dextran Sodium Sulfate-Induced Injury *in vivo*

**Figure 5:**
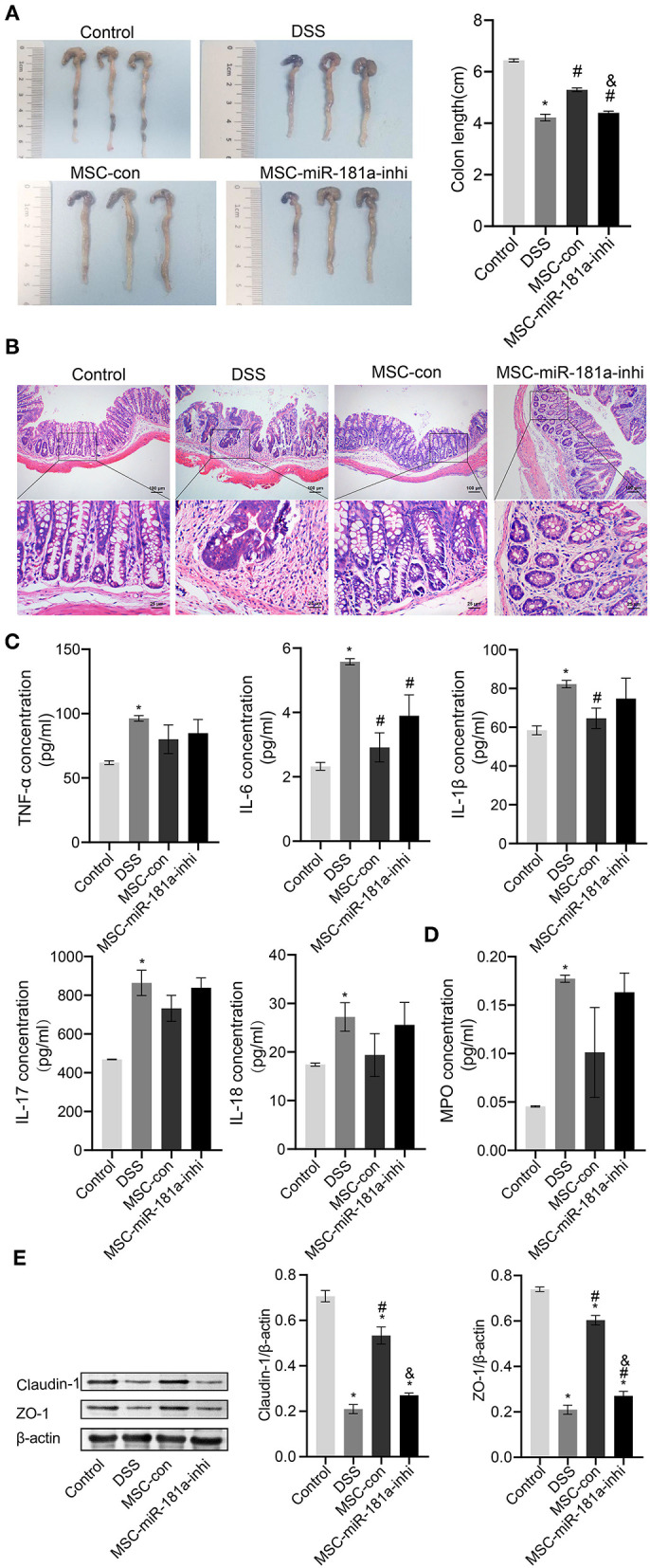
Exosomal miR-181a attenuated DSS-induced injury *in vivo*. **(A)** The appearance and length of the colon in mice were analyzed. **(B)** H&E staining of colonic pathological features in the mice. Scale bar: 100 or 25 μm. **(C)** The concentrations of TNF-α, IL-6, IL-1β, IL-17, and IL-18 in serum were measured *via* ELISA. **(D)** MPO activity in colon tissue was measured *via* ELISA. **(E)** Western blotting was used to detect the expression of the tight junction proteins Claudin-1 and ZO-1. *P < 0.05 vs. the control. ^#^*P* < 0.05 vs. the DSS group. ^&^*P* < 0.05 vs. the MSC-con group. *n* = 10 mice/group. DSS, dextran sodium sulfate; MPO, myeloperoxidase.

To further investigate whether the effects of MSC-Exos in colitis mice were related to miR-181a, the DSS-induced mice were injected with Exos *via* a caudal vein extracted from human MSCs (hMSCs) transfected with miR-181a inhibitor. The results showed that the colon length in the MSC-con group was longer than that in the DSS group, while this was reversed in the MSC-miR-181a-inhi group (Figure 5A). Furthermore, mice in the MSC-Con groups had less severe colitis by H&E staining than in the DSS group (Figure 5B). However, in the MSC-miR-181a-inhi group, the protection effect of MSC-miR-181a against colitis was partially reversed. These results suggested that miR-181a in MSC-Exos might play an important role in recovery from colitis. Compared with the DSS group, the serum levels of inflammatory cytokines, including TNF-α, IL-6, IL-17, and IL-18, were downregulated in the MSC-con group. Moreover, the effects were reversed in the MSC-miR-181a-inhi group. Among them, the changes in the IL-6 concentration were significant (Figure 5C). In addition, the MPO concentration in colon tissue in the MSC-con group and MSC-miR-181a-inhi group was downregulated compared with that in the DSS model group (Figure 5D). To further investigate the mechanism of MSC-Exo-mediated protection on intestinal barrier function, Western blotting demonstrated that the expression levels of Claudin-1 and ZO-1 were significantly increased in the MSC-con group compared with the DSS group (Figure 5E). However, in the MSC-miR-181a-inhi group, the above impacts were significantly reversed. These results indicated that the Exos extracted from MSCs with miR-181a silenced reversed the therapeutic effects of MSC-Exos in colitis mice *in vivo*.

### Exosomal microRNA-181a Rescued Lipopolysaccharide-Induced Damage *in vitro*

**Figure 6:**
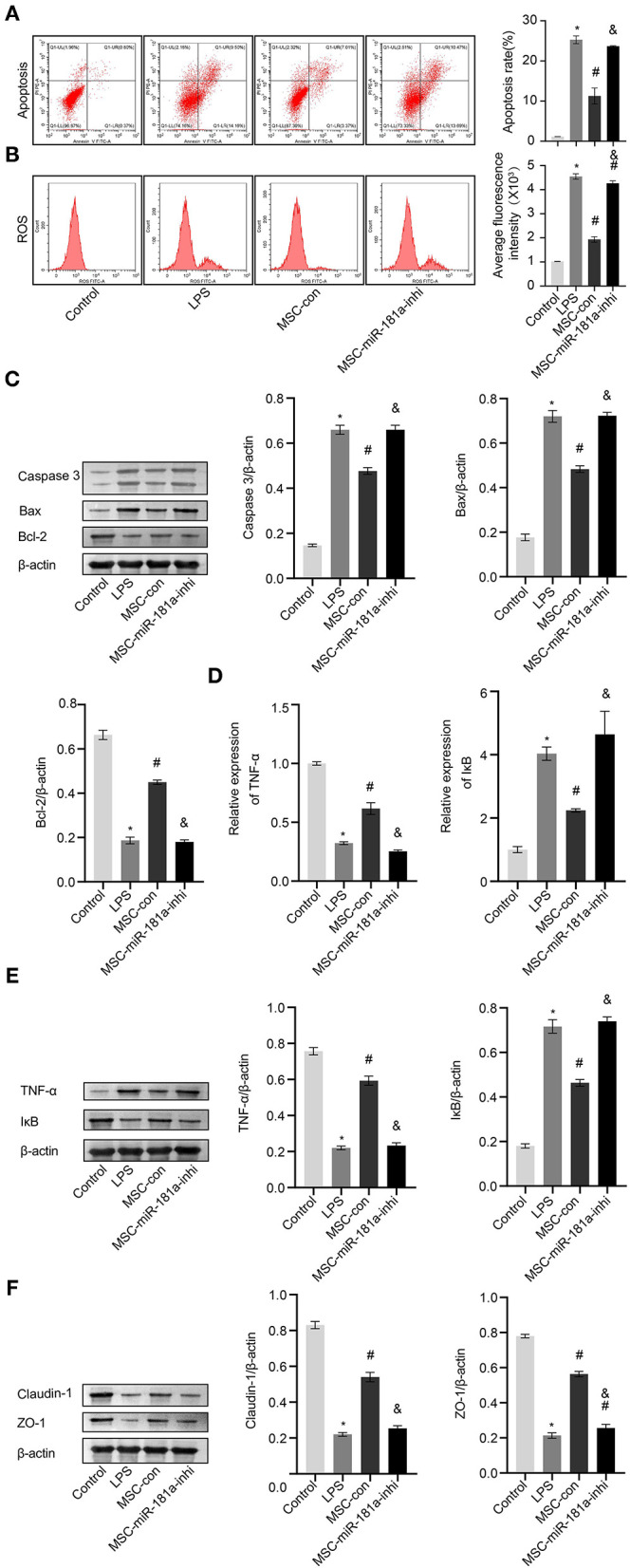
Exosomal miR-181a rescued LPS-induced damage *in vitro*. **(A)** HCOEPIC apoptosis was detected by flow cytometry. **(B)** Measurement of ROS in the HCOEPICs. **(C)** Western blotting was used to detect the expression levels of Caspase-3, Bax, and Bcl-2 in HCOEPICs. **(D)** qRT-PCR was used to detect the expression levels of TNF-α and IκB in HCOEPICs. **(E)** Western blotting was performed to detect the expression levels of TNF-α and IκB in HCOEPICs. **(F)** The expression levels of Claudin-1 and ZO-1 were detected *via* Western blotting. *P < 0.05 vs. the control. ^#^*P* < 0.05 vs. the LPS group. ^&^*P* < 0.05 vs. the MSC-con group. LPS, lipopolysaccharide; HCOEPIC, human colonic epithelial cell; ROS, reactive oxygen species.

To further study the role of MSC-derived exosomal miR-181a on LPS-induced HCOEPICs *in vitro*, flow cytometry assays were conducted. The apoptosis and ROS results showed that the apoptosis and oxidative stress levels in the MSC-con group were significantly inhibited compared with those in the LPS group. However, the miR-181a inhibitor exacerbated the apoptosis and oxidative stress levels in the cells, in contrast to the MSC-con group (Figures 6A,B). Furthermore, Western blotting analysis of apoptosis-related proteins showed that compared with those in the LPS group, the expression levels of Caspase-3 and Bax were significantly decreased and those of Bcl-2 were increased in the MSC-con group. Notably, the opposite impacts were observed in the MSC-miR-181a-inhi group (Figure 6C). We wondered whether Exos affect the TNF-α/IκB signaling pathway in LPS-induced colitis; therefore, qRT-PCR and Western blotting experiments were performed. The expression levels of TNF-α were significantly downregulated at both the transcriptional and translational levels in the MSC-miR-181a-inhi group, while those of IκB were upregulated, in contrast to those in the MSC-con group (Figures 6D,E). The Western blotting results further showed that the expression levels of Claudin-1 and ZO-1 were significantly reversed in the MSC-miR-181a-inhi group compared with the MSC-con group (Figure 6F). Altogether, these results indicated that exosomal miR-181a mediated protection against LPS-induced inflammation. In addition, the TNF-α/IκB signaling pathway might be involved in the protective effect of miR-181a.

### Effects of Mesenchymal Stem Cell-Derived Exosomes on the Gut Microbiota Composition in Dextran Sodium Sulfate-Induced Colitis Mice

**Figure 7:**
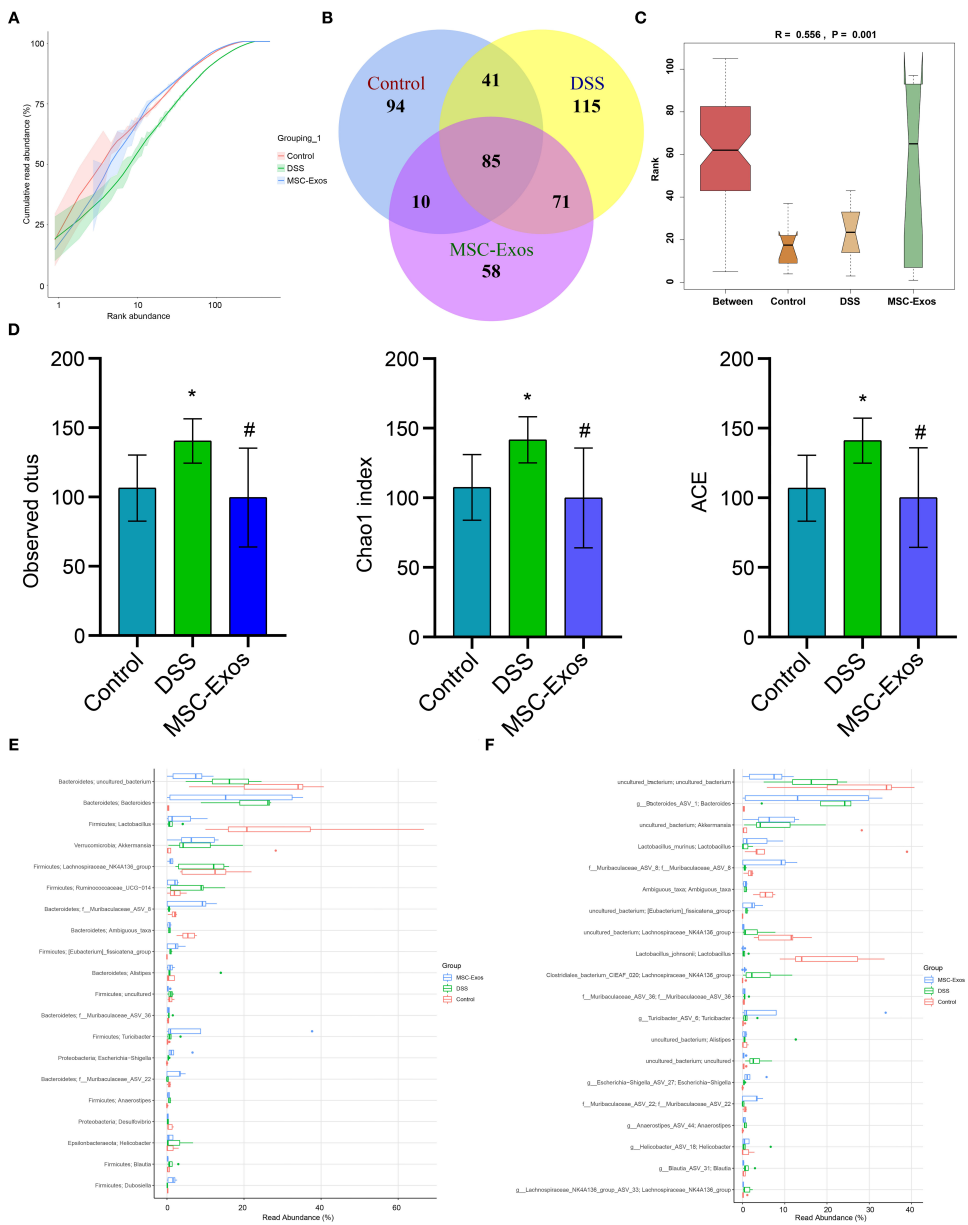
Changes in intestinal microbiota. **(A)** Rank-abundance graph showing the richness and uniformity of each group. **(B)** Venn diagram showing the results for OTUs. **(C)** ANOSIM analysis showing differences in community structure among the groups. *P* < 0.05 indicates statistical significance. **(D)** Alpha diversity was used to analyze changes in the observed OTUs and Chao1 and ACE indexes. **(E,F)** At the genus and species levels, the relative abundance of the microbiota in the samples, respectively. *P < 0.05 vs. the control. ^#^*P* < 0.05 vs. the DSS group. *n* = 5 mice/group. OTUs, operational taxonomic units; DSS, dextran sodium sulfate.

To further analyze whether MSC-Exos have effects on the gut microbiota structure in colitis mice, 16S rRNA sequencing was performed. The result of the rank-abundance graph showed that the richness and uniformity of each group of samples were eligible (Figure 7A). According to the results of operational taxonomic unit (OTU) cluster analysis, the Venn plot showed that the number of OTUs in the control group, DSS group, and MSC-Exo group was 230, 313, and 225, respectively, among which 85 were common OTUs (Figure 7B). ANOSIM analysis results showed the significant differences in community structure among the different groups (Figure 7C). Alpha diversity analysis showed that the observed OTUs, Chao1, and ACE indexes in the DSS group were higher than those in the control group. After treatment with MSC-Exos, we found that the indexes of observed OTUs, Chao1, and ACE were similar to those in the control group (Figure 7D). The relative abundance of species at the genus level showed the top 20 species, among which the expression abundance of *Lactobacillus* decreased in the DSS group, while the expression abundance of *Bacteroides* increased compared with the control group. After treatment with MSC-Exos, the changes in related bacteria were partially reversed. The expression abundance of *Lactobacillus* showed an upward trend, while that of *Bacteroides* showed a downward trend after treatment with MSC-Exos (Figure 7E). The relative abundance of species at the species level showed the top 20 species, among which the expression abundance of *Lactobacillus_murinus* decreased in the DSS group, while the expression abundance of g_*Bacteroides_ASV_1* increased compared with that of the control group. After treatment with MSC-Exos, the changes in related bacteria were partially reversed. The expression abundance of *Lactobacillus* showed an upward trend, while that of *Bacteroides* showed a downward trend after treatment with MSC-Exos (Figure 7F). These results suggested that MSC-Exo treatment can alleviate the disturbance in the gut microbiota induced by DSS.

## Discussion

It is urgent to study an effective treatment for UC. In our study, a DSS-induced colitis model was established *in vivo*. Through *in vivo* experiments, we found that intragastric administration of MSC-derived exosomal miR-181a increased the expression levels of colonic tight junction proteins and alleviated the symptoms of DSS-induced colitis in mice. In addition, the composition and structure of the gut microbiota were changed. By establishing an *in vitro* LPS-induced inflammation model of HCOEPIC, we found that MSC-derived exosomal miR-181a reduced LPS-induced apoptosis and oxidative stress in HCOEPICs. At the same time, the vitality of the HCOEPICs was increased. Our results suggested that MSC-derived exosomal miR-181a might ameliorate UC by affecting the gut microbiota and promoting intestinal barrier function.

Previous studies have shown that bone marrow MSC-derived EVs (BMSC-EVs) can protect against experimental colitis by downregulating proinflammatory cytokines, regulating oxidative stress balance, and reducing apoptosis (23). In this study, *in vivo* experiments showed that MSC-Exos alleviated inflammatory symptoms in colon tissue and reduced the concentration of inflammatory cytokines in the serum of colitis mice. MSC-Exos increased the viability of LPS-induced HCOEPICs and reduced apoptosis *in vitro*. In addition, LPS-stimulated MSC-Exos showed a better effect. The results of *in vitro* and *in vivo* experiments suggested that MSC-Exos had certain protective effects in experimental colitis, and the effects of stressed MSC-Exos were stronger. We speculated that MSCs might produce more Exos with therapeutic effects under LPS stimulation, thus playing a better therapeutic role in the treatment of UC. LPS could stimulate MSCs to increase the expression of miR-181a in MSC-Exos. The elevated miR-181a was transported to colonic epithelial cells through Exos, alleviating the inflammation of HCOEPICs stimulated by LPS and regulating the expression of related inflammatory pathways. The mechanism by which LPS stimulates the increase of miR-181a in the Exos of MSCs supernatant deserves further study. On the one hand, LPS-stimulated MSCs can simulate the inflammation model of UC. On the other hand, certain pretreatments, such as LPS, can improve the biological and functional properties of MSCs to a certain extent (24). In the study of cardiomyocyte injury, LPS stimulated the increase of miR-181a in MSCs and relieved the inflammation and oxidative stress of cardiomyocytes induced by H_2_O_2_ (25). Inspired by this article, we speculated that LPS stimulated miR-181a in MSC-Exos to have the same alleviating effects on UC.

MSC-Exos contain many miRNAs, including miR-181a (26). MiR-181a may not be the only mediator that may play a role in Exos. It has been reported that miR-181a plays an important role in immunity and inflammation regulation (27). In addition, miR-181a is downregulated in colorectal cancer (28). However, the mechanism of miR-181a in UC is not fully understood. In this study, GEO database analysis demonstrated that the expression of miR-181a in UC patients was significantly lower than that in normal controls. Moreover, the differential expression of miR-181a was the most significant. GO analysis showed that miR-181a might be related to the regulation of cellular amide metabolic processes, and KEGG analysis showed that miR-181a might be correlated with miRNAs in cancer. These results indicated that miR-181a might play an important role in UC. In summary, we chose miR-181a as our research focus. In future research, we will directly analyze MSC-Exos through microarray technology, screening out miRNAs or mRNAs that may alleviate UC, and conduct in-depth studies.

MiRNAs can be encapsulated in Exos and then transferred to target receptor cells to perform their biological functions (29). Our results verified high expression levels of miR-181a in hMSCs. MSC-Exos successfully inhibited the expression of miR-181a by transfection with miR-181a inhibitor. The use of lentivirus or Cas9 may be able to achieve a better silence effect, but due to time constraints, we did not study this. In the next experiment, we will use lentivirus, Cas9, and plasmid to silence miR-181a and screen and compare the best method for further study. Next, *in vivo* and *in vitro* experiments verified that silencing exosomal miR-181a aggravated inflammation in colon tissue and increased inflammatory factor levels and apoptosis. It has been reported that MPO might serve as an important diagnostic and prognostic tool in assessing UC status (30). The MPO activity in colon tissue was downregulated in the MSC-con group compared with that in the DSS group. This indicated that silencing miR-181a destroyed the therapeutic effects of MSC-Exos in DSS-induced colitis mice. It was suggested that MSC-derived exosomal miR-181a might have protective effects in UC. MSC-Exos might have more than one mediator that plays a role in the remission of UC. For example, metallothionein-2 in MSC-Exos has been shown to play a crucial role in alleviating DSS-induced mice (31). Due to funding and time constraints, we have not studied this in-depth. We look forward to studying and discovering more possible mechanisms in future experiments to lay the foundation for the development of targeted drugs for UC.

The presence of excess TNF-α has a significant effect on UC, and injection of a TNF-α inhibitor is a mature method for treatment of UC (32). In our study, when miRNA-181a was silenced, TNF-α expression was upregulated both *in vivo* and *in vitro*, while IκB, as a downstream pathway, was downregulated. Li et al. confirm that miRNA-181a downregulates the expression of related proteins by targeting TNF-α in adipocytes (33), which is consistent with our results. In addition, we predicted that miR-181a and TNF-α might have a corresponding targeting relationship through starbase 3.0. Therefore, we speculated that MSC-derived exosomal miR-181a might alleviate UC by targeting TNF-α. Due to time and funding constraints, we have not done a targeted verification of 3′UTR luciferase assay. In future studies, we will further study the targeting relationship between miR-181a and TNF-α.

The intestinal barrier is an important line of defense in the intestinal tract, and its dysfunction can disrupt immune homeostasis and cause a serious inflammatory response (34). UC patients have intestinal barrier dysfunction (35). Both Claudin-1 and ZO-1 are marker proteins of the intestinal barrier (36) and are important for maintaining intestinal function and integrity (37). The results of our animal and cell experiments suggest that MSC-derived exosomal miR-181a can also upregulate the expression of Claudin-1 and ZO-1 and improve intestinal barrier function.

The gut microbiota is closely related to UC. Studies have shown that extracellular polysaccharides (EPSs) reduce the risk of inflammatory bowel disease (IBD) symptoms by regulating the gut microbiota (38). In this study, 16S rRNA sequencing of the gut microbiota showed that MSC-derived exosomal miR-181a was beneficial to the richness and diversity of the gut microbiota in DSS-induced mice. Compared with those in the control group, the gut microbiota α-diversity and the composition of the gut microbiota in the DSS-induced mice were significantly changed. Among them, the expression abundance of *Lactobacillus* decreased in the DSS group, while the expression abundance of *Bacteroides* increased compared with those in the control group. The expression abundance of *Lactobacillus* showed an upward trend, while that of *Bacteroides* showed a downward trend after treatment with MSC-Exos. These results indicated that treatment with MSC-Exos could inhibit the colonization of pathogenic bacteria and promote that of probiotics. After treatment, the composition of the gut microbiota in the DSS-induced mice tended to develop normally, significantly improving experimental colitis. This was similar to the effects of *Scutellaria baicalensis* Georgi polysaccharide on the regulation of gut microbiota on UC (39). Due to time constraints, the effects of inhibiting miR-181a expression in Exos on the gut microbiota have not been studied. In the next study, we will collect the feces of the control, DSS, MSC-Exo-mimics, and MSC-Exo-inhibitor groups. The mice in the normal group and the model group were treated as described in this study. The MSC-Exo-mimics group and MSC-Exo-inhibitor groups were injected with tail vein Exos extracted from MSCs transfected with mimics and inhibitors, respectively. 16S rRNA technology was used to detect the enrichment of gut microbiota in each group.

Our data suggested that MSC-derived exosomal miR-181a played a preventive role in the experimental model of DSS, at least in part by promoting the gut microbiota and intestinal barrier function. This indicates that MSC-derived exosomal miR-181a is a promising method for the treatment of UC. The regulation of intestinal barrier function by MSC-derived exosomal miR-181a was preliminarily confirmed, while the effects of the gut microbiota on UC remain unclear and need to be further studied. In addition, a mouse animal was employed in this study. However, whether MSC-derived exosomal miR-181a plays a role in humans remains to be further studied.

## Conclusion

Our study demonstrated that Exos derived from MSCs might relieve the inflammation in DSS-induced colitis model mice and protect the intestinal barrier function *via* transporting miR-181a *in vivo*. The study verified that Exos derived from MSCs alleviated LPS-induced epithelial cell inflammation and upregulated tight junction protein expression *via* transporting miR-181a *in vitro*. In addition, the protective effects of MSC-Exos on DSS-induced colitis might be related to the regulation of gut microbiota. This study provided new clues for the treatment of UC and laid a foundation for the study of the mechanism *via* which MSC-derived exosomal miR-181a improves UC.

## Data Availability Statement

The datasets presented in this study can be found in online repositories. The names of the repository/repositories and accession number(s) can be found below: https://www.ncbi.nlm.nih.gov/ (PRJNA699385).

## Ethics Statement

The animal study was reviewed and approved by the Animal Ethics Committee at The Second Xiangya Hospital of Central South University.

## Author Contributions

LG designed this study and wrote the manuscript. FR and SW revised the manuscript and participated in data analysis. XF, LY, and GL participated in the experiments. All authors read and approved the final manuscript.

## Conflict of Interest

The authors declare that the research was conducted in the absence of any commercial or financial relationships that could be construed as a potential conflict of interest.

## Supplementary Material

Supplementary Figure 1 Validation of MSC-derived Exos. **(A)** The structure of Exos was observed *via* TEM. **(B)** Nanoparticle Tracking Analysis was used to identify exosomal diameter. **(C)** The expression levels of TSG101, CD81, and CD63 were detected *via* Western blotting. **(D)** The absorbance of MSC-derived Exos by HCOEPICs. **(E)** The expression of MSC-derived exosome miR-181a was detected by qRT-PCR. **(F)** qRT-PCR was used to detect the expression levels of MSC-derived exosomal miR-181a after transfection with miR-181a inhibitor. ^*^*P* < 0.05, vs. the Control. ^#^*P* < 0.05, vs. the MSC-Exos group.

## References

[1] Ungaro R Mehandru S Allen PB Peyrin-Biroulet L Colombel JF. Ulcerative colitis. Lancet. (2017) 389:1756–70. 10.1016/S0140-6736(16)32126-2 27914657 PMC6487890

[2] Ng SC Shi HY Hamidi N Underwood FE Tang W Benchimol EI. Worldwide incidence and prevalence of inflammatory bowel disease in the 21st century: a systematic review of population-based studies. Lancet. (2018) 390:2769–78. 10.1016/S0140-6736(17)32448-0 29050646

[3] Kaplan GG. The global burden of IBD: from 2015 to 2025. Nat Rev Gastroenterol Hepatol. (2015) 12:720–7. 10.1038/nrgastro.2015.150 26323879

[4] Kern I Schoffer O Kiess W Henker J Laaß MW Winkler U. Incidence trends of pediatric onset inflammatory bowel disease in the years 2000-2009 in Saxony, Germany-first results of the Saxon Pediatric IBD Registry. PLoS One. (2021) 16:e0243774. 10.1371/journal.pone.0243774 33395450 PMC7781364

[5] Su HJ Chiu YT Chiu CT Lin YC Wang CY Hsieh JY. Inflammatory bowel disease and its treatment in 2018: Global and Taiwanese status updates. J Formos Med Assoc. (2019) 118:1083–92. 10.1016/j.jfma.2018.07.005 30054112

[6] Dave M Purohit T Razonable R Loftus EV Jr. Opportunistic infections due to inflammatory bowel disease therapy. Inflamm Bowel Dis. (2014) 20:196–212. 10.1097/MIB.0b013e3182a827d2 24051931

[7] Quezada SM McLean LP Cross RK. Adverse events in IBD therapy: the 2018 update. Expert Rev Gastroenterol Hepatol. (2018) 12:1183–91. 10.1080/17474124.2018.1545574 30791788

[8] Gonzalez-Pujana A Igartua M Santos-Vizcaino E Hernandez RM. Mesenchymal stromal cell based therapies for the treatment of immune disorders: recent milestones and future challenges. Expert Opin Drug Deliv. (2020) 17:189–200. 10.1080/17425247.2020.1714587 31918562

[9] Ko JZ Johnson S Dave M. Efficacy and safety of mesenchymal stem/stromal cell therapy for inflammatory bowel diseases: an up-to-date systematic review. Biomolecules. (2021) 11:82. 10.3390/biom11010082 33440772 PMC7827559

[10] Shi X Chen Q Wang F. Mesenchymal stem cells for the treatment of ulcerative colitis: a systematic review and meta-analysis of experimental and clinical studies. Stem Cell Res Ther. (2019) 10:266. 10.1186/s13287-019-1336-4 31443677 PMC6708175

[11] Volarevic V Markovic BS Gazdic M Volarevic A Jovicic N Arsenijevic N. Ethical and safety issues of stem cell-based therapy. Int J Med Sci. (2018) 15:36–45. 10.7150/ijms.21666 29333086 PMC5765738

[12] Kalluri R LeBleu VS. The biology, function, and biomedical applications of exosomes. Science. (2020) 367:eaau6977. 10.1126/science.aau6977 32029601 PMC7717626

[13] Chang YH Wu KC Harn HJ Lin SZ Ding DC. Exosomes and stem cells in degenerative disease diagnosis and therapy. Cell Transplant. (2018) 27:349–63. 10.1177/0963689717723636 29692195 PMC6038041

[14] Li Y Altemus J Lightner AL. Mesenchymal stem cells and acellular products attenuate murine induced colitis. Stem Cell Res Ther. (2020) 11:515. 10.1186/s13287-020-02025-7 33256827 PMC7706051

[15] Ma ZJ Wang YH Li ZG Wang Y Li BY Kang HY. Immunosuppressive effect of exosomes from mesenchymal stromal cells in defined medium on experimental colitis. Int J Stem Cells. (2019) 12:440–8. 10.15283/ijsc18139 31242720 PMC6881044

[16] Doyle LM Wang MZ. Overview of extracellular vesicles, their origin, composition, purpose, and methods for exosome isolation and analysis. Cells. (2019) 8:727. 10.3390/cells8070727 31311206 PMC6678302

[17] Knarr M Avelar RA Sekhar SC Kwiatkowski LJ Dziubinski ML McAnulty J. miR-181a initiates and perpetuates oncogenic transformation through the regulation of innate immune signaling. Nat Commun. (2020) 11:3231. 10.1038/s41467-020-17030-w 32591511 PMC7320168

[18] Liu X Luo M Meng H Zeng Q Xu L Hu B. MiR-181a regulates CD4(+) T cell activation and differentiation by targeting IL-2 in the pathogenesis of myasthenia gravis. Eur J Immunol. (2019) 10.1002/eji.20184800731348521

[19] Qiu L Chen W Wu C Yuan Y Li Y. Exosomes of oral squamous cell carcinoma cells containing miR-181a-3p induce muscle cell atrophy and apoptosis by transmissible endoplasmic reticulum stress signaling. Biochem Biophys Res Commun. (2020) 533:831–7. 10.1016/j.bbrc.2020.09.066 32998818

[20] Yan Y Jiang W Tan Y Zou S Zhang H Mao F. hucMSC Exosome-Derived GPX1 is required for the recovery of hepatic oxidant injury. Mol Ther. (2017) 25:465–79. 10.1016/j.ymthe.2016.11.019 28089078 PMC5368592

[21] Li L Wan G Han B Zhang Z. Echinacoside alleviated LPS-induced cell apoptosis and inflammation in rat intestine epithelial cells by inhibiting the mTOR/STAT3 pathway. Biomed Pharmacother. (2018) 104:622–8. 10.1016/j.biopha.2018.05.072 29803175

[22] Wu XX Huang XL Chen RR Li T Ye HJ Xie W. Paeoniflorin prevents intestinal barrier disruption and inhibits lipopolysaccharide (lps)-induced inflammation in CaCo-2 cell monolayers. Inflammation. (2019) 42:2215–25. 10.1007/s10753-019-01085-z 31473900

[23] Yang J Liu XX Fan H Tang Q Shou ZX Zuo DM. Extracellular vesicles derived from bone marrow mesenchymal stem cells protect against experimental colitis *via* attenuating colon inflammation, oxidative stress and apoptosis. PLoS One. (2015) 10:e0140551. 10.1371/journal.pone.0140551 26469068 PMC4607447

[24] Zhu J Lu K Zhang N Zhao Y Ma Q Shen J. Myocardial reparative functions of exosomes from mesenchymal stem cells are enhanced by hypoxia treatment of the cells *via* transferring microRNA-210 in an nSMase2-dependent way. Artif Cells Nanomed Biotechnol. (2018) 46:1659–70. 10.1080/21691401.2017.1388249 29141446 PMC5955787

[25] Liu HY Yu LF Zhou TG Wang YD Sun DH Chen HR. Lipopolysaccharide-stimulated bone marrow mesenchymal stem cells-derived exosomes inhibit H_2_O_2_-induced cardiomyocyte inflammation and oxidative stress *via* regulating miR-181a-5p/ATF2 axis. Eur Rev Med Pharmacol Sci. (2020) 24:10069–77. 10.26355/eurrev_202010_23224 33090414

[26] Phinney DG Pittenger MF. Concise review: MSC-derived exosomes for cell-free therapy. Stem Cells. (2017) 35:851–8. 10.1002/stem.2575 28294454

[27] Sonkoly E Ståhle M Pivarcsi A. MicroRNAs and immunity: novel players in the regulation of normal immune function and inflammation. Semin Cancer Biol. (2008) 18:131–40. 10.1016/j.semcancer.2008.01.005 18291670

[28] Kanaan Z Rai SN Eichenberger MR Barnes C Dworkin AM Weller C. Differential microRNA expression tracks neoplastic progression in inflammatory bowel disease-associated colorectal cancer. Hum Mutat. (2012) 33:551–60. 10.1002/humu.22021 22241525 PMC4410875

[29] Wani S Man Law IK Pothoulakis C. Role and mechanisms of exosomal miRNAs in IBD pathophysiology. Am J Physiol Gastrointest Liver Physiol. (2020) 319:G646–G54. 10.1152/ajpgi.00295.2020 33026230 PMC7792667

[30] Qian B Wang C Zeng Z Ren Y Li D Song JL. Ameliorative effect of sinapic acid on dextran sodium sulfate- (DSS-) induced ulcerative colitis in Kunming (KM) mice. Oxid Med Cell Longev. (2020) 2020:8393504. 10.1155/2020/8393504 33312339 PMC7719534

[31] Liu H Liang Z Wang F Zhou C Zheng X Hu T. Exosomes from mesenchymal stromal cells reduce murine colonic inflammation *via* a macrophage-dependent mechanism. JCI Insight. (2019) 4:e131273. 10.1172/jci.insight.131273 31689240 PMC6975270

[32] Kobayashi T Siegmund B Le Berre C Wei SC Ferrante M Shen B. Ulcerative colitis. Nat Rev Dis Primers. (2020) 6:74. 10.1038/s41572-020-0205-x 32913180

[33] Li H Chen X Guan L Qi Q Shu G Jiang Q. MiRNA-181a regulates adipogenesis by targeting tumor necrosis factor-α (TNF-α) in the porcine model. PLoS One. (2013) 8:e71568. 10.1371/journal.pone.0071568 24098322 PMC3787936

[34] Anderson RC Bassett SA Haggarty NW Gopal PK Armstrong KM Roy NC. Short communication: early-lactation, but not mid-lactation, bovine lactoferrin preparation increases epithelial barrier integrity of Caco-2 cell layers. J Dairy Sci. (2017) 100:886–91. 10.3168/jds.2016-11803 27939537

[35] Vancamelbeke M Vanuytsel T Farré R Verstockt S Ferrante M Van Assche G. Genetic and transcriptomic bases of intestinal epithelial barrier dysfunction in inflammatory bowel disease. Inflamm Bowel Dis. (2017) 23:1718–29. 10.1097/MIB.0000000000001246 28885228 PMC6461205

[36] Furuse M Sasaki H Tsukita S. Manner of interaction of heterogeneous claudin species within and between tight junction strands. J Cell Biol. (1999) 147:891–903. 10.1083/jcb.147.4.891 10562289 PMC2156154

[37] Lee SH. Intestinal permeability regulation by tight junction: implication on inflammatory bowel diseases. Intest Res. (2015) 13:11–8. 10.5217/ir.2015.13.1.11 25691839 PMC4316216

[38] Min Z Xiaona H Aziz T Jian Z Zhennai Y. Exopolysaccharides from *Lactobacillus plantarum* YW11 improve immune response and ameliorate inflammatory bowel disease symptoms. Acta Biochim Pol. (2020) 67:485–93. 10.18388/abp.2020_5171 33332076

[39] Cui L Guan X Ding W Luo Y Wang W Bu W. *Scutellaria baicalensis* Georgi polysaccharide ameliorates DSS-induced ulcerative colitis by improving intestinal barrier function and modulating gut microbiota. Int J Biol Macromol. (2021) 166:1035–45. 10.1016/j.ijbiomac.2020.10.259 33157130

