# Supplementary figures and images for: Exosomal MicroRNA-181a Derived From Mesenchymal Stem Cells Improves Gut Microbiota Composition, Barrier Function, and Inflammatory Status in an Experimental Colitis Model
Source: Front Med (Lausanne). 2021 Jun 24;8:660614. doi: 10.3389/fmed.2021.660614 (PMC8264068; doi:10.3389/fmed.2021.660614)

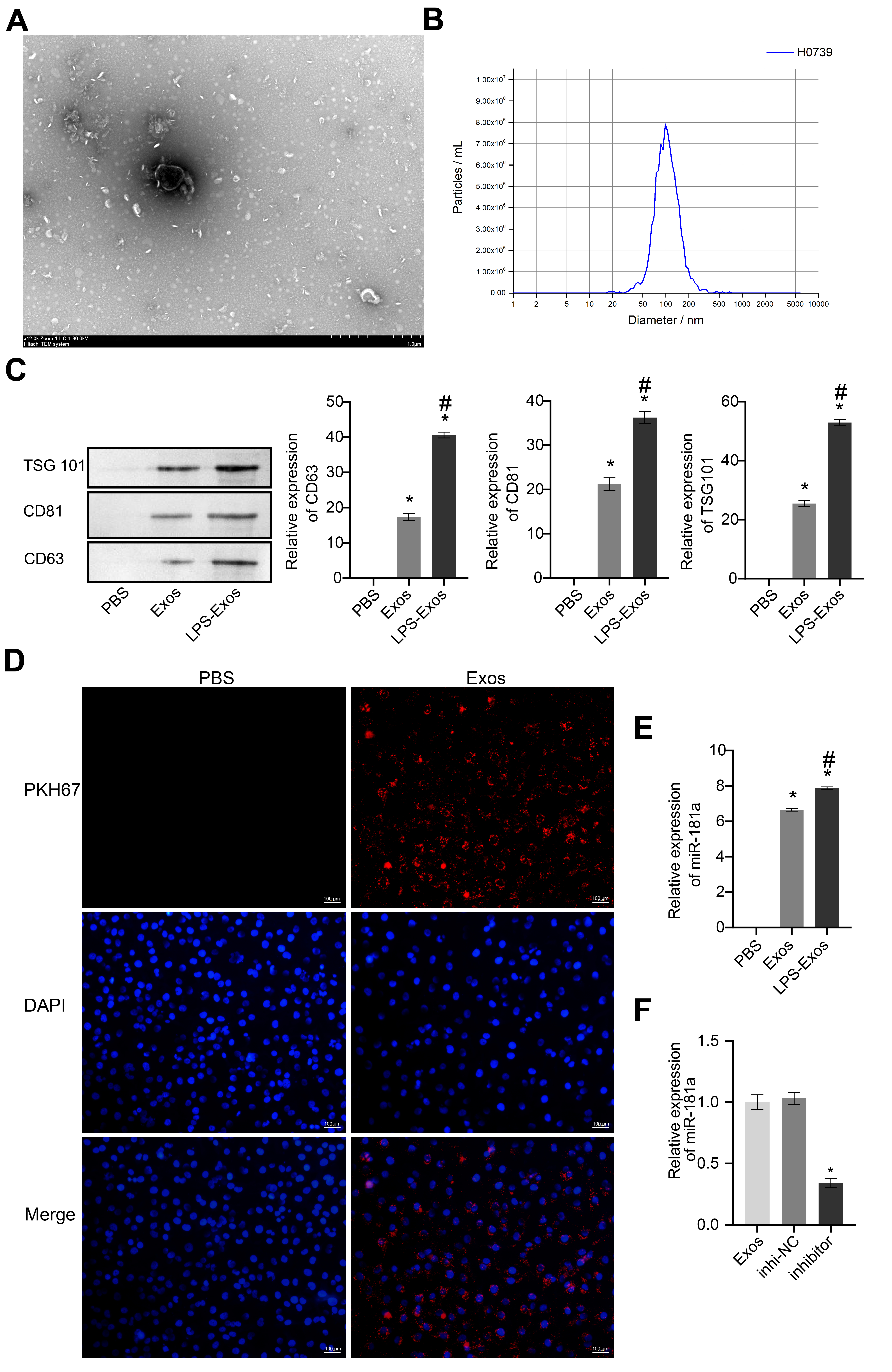

Supplement: Supplementary Figure 1 — Validation of MSC-derived Exos. (A) The structure of Exos was observed via TEM. (B) Nanoparticle Tracking Analysis was used to identify exosomal diameter. (C) The expression levels of TSG101, CD81, and CD63 were detected via Western blotting. (D) The absorbance of MSC-derived Exos by HCOEPICs. (E) The expression of MSC-derived exosome miR-181a was detected by qRT-PCR. (F) qRT-PCR was used to detect the expression levels of MSC-derived exosomal miR-181a after transfection with miR-181a inhibitor. *P < 0.05, vs. the Control. #P < 0.05, vs. the MSC-Exos group. [file Image_1.JPEG]
